# Triaqua­(1,4,7-triaza­cyclo­nonane-κ^3^ *N* ^1^,*N* ^4^,*N* ^7^)nickel(II) bromide nitrate

**DOI:** 10.1107/S160053681001620X

**Published:** 2010-05-08

**Authors:** Changchun Wen, Jianqi Lu, Zhong Zhang

**Affiliations:** a College of Chemistry and Chemical Engineering, Guangxi Normal University, Yucai Road 15, Guilin 541004, People’s Republic of China

## Abstract

In the title half-sandwich compound, [Ni(C_6_H_15_N_3_)(H_2_O)_3_]Br(NO_3_), the central Ni^II^ ion, lying on a threefold rotation axis, is six-coordinated by three amine N atoms from the face-capping triaza macrocycle and three water O atoms in a slightly distorted octa­hedral geometry. In the crystal, O—H⋯O hydrogen bonding and weak O—H⋯Br inter­actions associate the Ni^II^ cations and the counter-ions into a three-dimensional supra­molecular network.

## Related literature

For the preparation of 1,4,7-triaza­cyclo­nonane trihydro­bromide, see: Koyama & Yoshino (1972 ▶). For the applications of metal complexes containing 1,4,7-triaza­cyclo­nonane as small-mol­ecule models of metalloenzymes and metalloproteins and as mol­ecule-based magnets, see: Berseth *et al.* (2000 ▶); Chaudhury *et al.* (1985 ▶); Cheng *et al.* (2004 ▶); Deal *et al.* (1996 ▶); Hegg & Burstyn (1995 ▶); Hegg *et al.* (1997 ▶); Lin *et al.* (2001 ▶); Poganiuch *et al.* (1991 ▶); Williams *et al.* (1999 ▶). For related Ni^II^ complexes with 1,4,7-triaza­cyclo­nonane, see: Bencini *et al.* (1990 ▶); Stranger *et al.* (1992 ▶); Wang *et al.* (2003 ▶, 2005 ▶); Zompa & Margulis (1978 ▶).
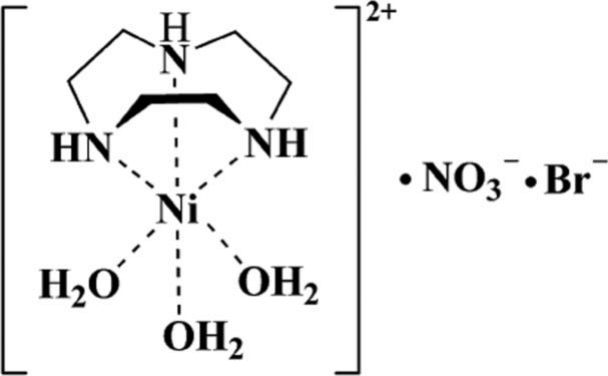


## Experimental

### 

#### Crystal data

[Ni(C_6_H_15_N_3_)(H_2_O)_3_]Br(NO_3_)
*M* *_r_* = 383.89
Cubic, 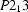

*a* = 11.300 (1) Å
*V* = 1442.9 (3) Å^3^
*Z* = 4
Mo *K*α radiation
μ = 4.14 mm^−1^
*T* = 298 K
0.29 × 0.27 × 0.18 mm

#### Data collection

Bruker APEXII CCD area-detector diffractometer
Absorption correction: multi-scan (*SADABS*; Bruker, 1998 ▶) *T*_min_ = 0.320, *T*_max_ = 0.480
15223 measured reflections
1110 independent reflections
985 reflections with *I* > 2σ(*I*)
*R*_int_ = 0.080

#### Refinement

*R*[*F* ^2^ > 2σ(*F* ^2^)] = 0.035
*wR*(*F* ^2^) = 0.072
*S* = 1.03
1110 reflections
61 parameters
H atoms treated by a mixture of independent and constrained refinement
Δρ_max_ = 0.36 e Å^−3^
Δρ_min_ = −0.47 e Å^−3^
Absolute structure: Flack (1983 ▶), 475 Friedel pairs
Flack parameter: 0.01 (3)

### 

Data collection: *APEX2* (Bruker, 2002 ▶); cell refinement: *SAINT* (Bruker, 2002 ▶); data reduction: *SAINT*; program(s) used to solve structure: *SHELXS97* (Sheldrick, 2008 ▶); program(s) used to refine structure: *SHELXL97* (Sheldrick, 2008 ▶); molecular graphics: *SHELXTL* (Sheldrick, 2008 ▶); software used to prepare material for publication: *SHELXTL*.

## Supplementary Material

Crystal structure: contains datablocks I, global. DOI: 10.1107/S160053681001620X/pb2027sup1.cif

Structure factors: contains datablocks I. DOI: 10.1107/S160053681001620X/pb2027Isup2.hkl

Additional supplementary materials:  crystallographic information; 3D view; checkCIF report

## Figures and Tables

**Table 1:** Hydrogen-bond geometry (Å, °)

*D*—H⋯*A*	*D*—H	H⋯*A*	*D*⋯*A*	*D*—H⋯*A*
O1—H4*A*⋯O2^i^	0.84 (4)	1.95 (5)	2.776 (5)	162 (4)
O1—H4*B*⋯Br1^ii^	0.85 (5)	2.48 (5)	3.312 (3)	167 (4)

Symmetry codes: (i) 
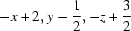
; (ii) 
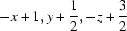
.

**Fig. 1.**
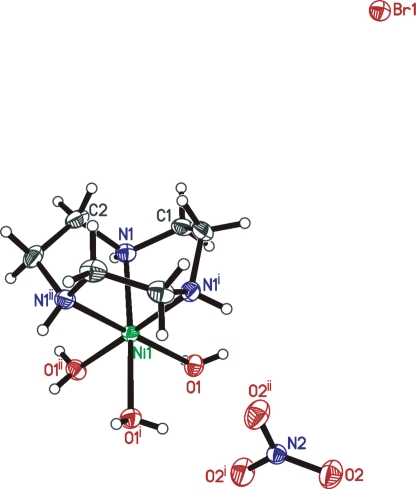
An ORTEP plot for the title compound (I) with the atom labelling scheme and 30% displacement ellipsoids. Symmetry codes: (i) y+1/2, -z+3/2, -x+2; (ii) -z+2, x-1/2, -y+3/2.

**Fig. 2.**
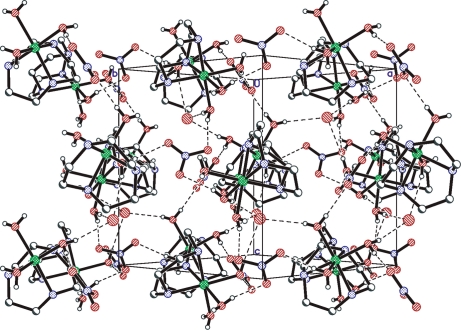
A view of the packing diagram of the title compound (I), showing the hydrogen–bonding supramolecular network. Hydrogen bonds are drawn in dashed lines. H atoms not involved in hydrogen bonds are omitted for clarity.

## supplementary crystallographic information

### Comment

The coordination chemistry of 1,4,7–triazacyclononane (TACN) has been extensively studied for its applications in the simulation of metalloenzymes and metalloproteins (Chaudhury *et al.*, 1985; Deal *et al.*, 1996; Hegg & Burstyn, 1995; Hegg *et al.*, 1997; Lin *et al.*, 2001; Williams *et al.*, 1999) as well as in constructing molecule–based magnetic materials (Berseth *et al.*, 2000; Cheng *et al.*, 2004; Poganiuch *et al.*, 1991). In general, TACN ligand can form stable sandwich complexes with many transition metals (Stranger *et al.*, 1992; Zompa & Margulis, 1978) or functions as a terminal chelator for the assembly of binuclear/polynuclear species and coordination polymers supported by bridging ligands (Bencini *et al.*, 1990; Wang *et al.*, 2005; Wang *et al.*, 2003). In this paper, a half–sandwich type Ni^II^ complex with TACN has been synthesized and characterized.

In the selected crystal, the title compound (I) crystallizes in a chiral space group *P2_1_3* and Flack parameter of 0.01 (3) indicates that a spontaneous resolution has been achieved during crystallization. As depicted in Fig. 1, the Ni^II^ center in the complex cation lies on a three–fold rotation axis and three amine N atoms from facially coordinated TACN and three water molecules complete the slightly distorted octahedral arrangement. Upon coordination, three five–membered Ni—N—C—C—N chelating rings subtended at metal center adopt (λλλ) conformation, which is the source of the chirality of the crystal. Ni—N [2.091 (3) Å] and Ni—O [2.089 (3) Å] bond lengths are both in the normal ranges, meanwhile N—Ni—N bond angle is smaller than that of O—Ni—O due to the small size of TACN ring. Counter–ions NO_3_^-^ and Br^-^ interconnect neighbouring cations by O—H···O hydrogen bond and O—H···Br^-^ weak interaction (Table 1) into three–dimensional supramolecular network (Fig. 2).

### Experimental

1,4,7–Triazacyclononane trihydrobromide (TACN^.^3HBr) was prepared by following a modified published method (Koyama & Yoshino, 1972).

To a solution of 0.074 g (0.02 mmol) of TACN^.^3HBr in water (10 ml), 0.1 M NaOH was added to adjust the pH to 6. Then aqueous solution (5 ml) of 0.058 g (0.02 mmol) of Ni(NO_3_)_2_^.^6H_2_0 was added and the resulting mixture was stirred under reflux for 6 h. After cooling, the mixture was filtered, and the filtrate was allowed to standing at ambient temperature. Plate–like green single crystals suitable for X–ray crystallographic analysis were collected by slow evaporation of the filtrate within two months.

### Refinement

All methylene H atoms were placed at calculated positions and refined as riding on their parent atoms [C—H = 0.97 Å and *U*_iso_(H) = 1.2 *U*_eq_(C)]. The H atoms of amine groups and water molecules were located in a difference Fourier map as riding atoms, with *U*_iso_(H) = 1.5 *U*_eq_(N) and 1.5 *U*_eq_(O).

### Crystal data

**Table:**

[Ni(C_6_H_15_N_3_)(H_2_O)_3_]Br(NO_3_)	*D*_x_ = 1.767 Mg m^−^^3^
*M**_r_* = 383.89	Mo *K*α radiation, λ = 0.71073 Å
Cubic, *P*2_1_3	Cell parameters from 13409 reflections
Hall symbol: P 2ac 2ab 3	θ = 3.1–27.4°
*a* = 11.300 (1) Å	µ = 4.14 mm^−^^1^
*V* = 1442.9 (3) Å^3^	*T* = 298 K
*Z* = 4	Plate, green
*F*(000) = 784	0.29 × 0.27 × 0.18 mm

### Data collection

**Table:**

Bruker APEXII CCD area-detector diffractometer	1110 independent reflections
Radiation source: fine-focus sealed tube	985 reflections with *I* > 2σ(*I*)
graphite	*R*_int_ = 0.080
φ and ω scans	θ_max_ = 27.4°, θ_min_ = 3.1°
Absorption correction: multi-scan (*SADABS*; Bruker, 1998)	*h* = −14→14
*T*_min_ = 0.320, *T*_max_ = 0.480	*k* = −14→14
15223 measured reflections	*l* = −14→14

### Refinement

**Table:**

Refinement on *F*^2^	Secondary atom site location: difference Fourier map
Least-squares matrix: full	Hydrogen site location: inferred from neighbouring sites
*R*[*F*^2^ > 2σ(*F*^2^)] = 0.035	H atoms treated by a mixture of independent and constrained refinement
*wR*(*F*^2^) = 0.072	*w* = 1/[σ^2^(*F*_o_^2^) + (0.0232*P*)^2^ + 1.6516*P*] where *P* = (*F*_o_^2^ + 2*F*_c_^2^)/3
*S* = 1.03	(Δ/σ)_max_ < 0.001
8717 reflections	Δρ_max_ = 0.36 e Å^−^^3^
61 parameters	Δρ_min_ = −0.46 e Å^−^^3^
0 restraints	Absolute structure: Flack (1983), 475 Friedel pairs
Primary atom site location: structure-invariant direct methods	Flack parameter: 0.01 (3)

### Special details

**Table:**

Geometry. All esds (except the esd in the dihedral angle between two l.s. planes) are estimated using the full covariance matrix. The cell esds are taken into account individually in the estimation of esds in distances, angles and torsion angles; correlations between esds in cell parameters are only used when they are defined by crystal symmetry. An approximate (isotropic) treatment of cell esds is used for estimating esds involving l.s. planes.
Refinement. Refinement of *F*^2^ against ALL reflections. The weighted *R*-factor wR and goodness of fit *S* are based on *F*^2^, conventional *R*-factors *R* are based on *F*, with *F* set to zero for negative *F*^2^. The threshold expression of *F*^2^ > σ(*F*^2^) is used only for calculating *R*-factors(gt) etc. and is not relevant to the choice of reflections for refinement. *R*-factors based on *F*^2^ are statistically about twice as large as those based on *F*, and *R*- factors based on ALL data will be even larger.

### Fractional atomic coordinates and isotropic or equivalent isotropic displacement parameters (Å^2^)

**Table:**

	*x*	*y*	*z*	*U*_iso_*/*U*_eq_	
Ni1	1.06169 (4)	0.56169 (4)	0.93831 (4)	0.02729 (19)	
Br1	0.25347 (4)	0.24653 (4)	0.75347 (4)	0.0437 (2)	
C1	0.8566 (4)	0.4233 (4)	0.8872 (4)	0.0437 (11)	
H1A	0.8123	0.3498	0.8858	0.052*	
H1B	0.8438	0.4636	0.8125	0.052*	
C2	1.0118 (4)	0.3128 (4)	0.9995 (4)	0.0449 (11)	
H2A	1.0326	0.2365	0.9660	0.054*	
H2B	0.9417	0.3022	1.0478	0.054*	
N1	0.9850 (3)	0.3973 (3)	0.9019 (3)	0.0353 (8)	
H3	1.0226	0.3631	0.8407	0.053*	
N2	0.9466 (3)	0.9466 (3)	0.9466 (3)	0.0316 (11)	
O1	1.0196 (3)	0.6447 (3)	0.7786 (3)	0.0391 (8)	
O2	0.8854 (3)	1.0345 (3)	0.9196 (3)	0.0577 (9)	
H4B	0.947 (5)	0.659 (4)	0.765 (4)	0.050 (14)*	
H4A	1.040 (4)	0.598 (4)	0.723 (4)	0.050 (14)*	

### Atomic displacement parameters (Å^2^)

**Table:**

	*U*^11^	*U*^22^	*U*^33^	*U*^12^	*U*^13^	*U*^23^
Ni1	0.02729 (19)	0.02729 (19)	0.02729 (19)	−0.0011 (2)	0.0011 (2)	0.0011 (2)
Br1	0.0437 (2)	0.0437 (2)	0.0437 (2)	−0.0012 (2)	0.0012 (2)	−0.0012 (2)
C1	0.041 (2)	0.042 (3)	0.049 (3)	−0.016 (2)	−0.009 (2)	0.005 (2)
C2	0.054 (3)	0.027 (2)	0.054 (3)	−0.0022 (19)	0.010 (2)	0.0063 (19)
N1	0.0365 (19)	0.0349 (19)	0.0346 (19)	−0.0022 (14)	0.0050 (14)	−0.0021 (14)
N2	0.0316 (11)	0.0316 (11)	0.0316 (11)	0.0002 (15)	0.0002 (15)	0.0002 (15)
O1	0.0424 (18)	0.0419 (18)	0.0330 (18)	0.0052 (14)	−0.0007 (13)	0.0024 (12)
O2	0.055 (2)	0.054 (2)	0.065 (2)	0.0156 (16)	0.0149 (17)	0.0133 (18)

### Geometric parameters (Å, °)

**Table:**

Ni1—O1^i^	2.089 (3)	C2—N1	1.490 (5)
Ni1—O1	2.089 (3)	C2—C1^ii^	1.520 (6)
Ni1—O1^ii^	2.089 (3)	C2—H2A	0.9700
Ni1—N1	2.091 (3)	C2—H2B	0.9700
Ni1—N1^ii^	2.091 (3)	N1—H3	0.8987
Ni1—N1^i^	2.091 (3)	N2—O2^iii^	1.248 (3)
C1—N1	1.490 (6)	N2—O2^iv^	1.248 (3)
C1—C2^i^	1.520 (6)	N2—O2	1.248 (3)
C1—H1A	0.9700	O1—H4B	0.85 (5)
C1—H1B	0.9700	O1—H4A	0.84 (4)
			
O1^i^—Ni1—O1	84.90 (14)	H1A—C1—H1B	108.1
O1^i^—Ni1—O1^ii^	84.90 (14)	N1—C2—C1^ii^	111.7 (3)
O1—Ni1—O1^ii^	84.90 (14)	N1—C2—H2A	109.3
O1^i^—Ni1—N1	177.00 (13)	C1^ii^—C2—H2A	109.3
O1—Ni1—N1	97.72 (13)	N1—C2—H2B	109.3
O1^ii^—Ni1—N1	93.87 (12)	C1^ii^—C2—H2B	109.3
O1^i^—Ni1—N1^ii^	93.87 (12)	H2A—C2—H2B	108.0
O1—Ni1—N1^ii^	177.00 (13)	C1—N1—C2	114.0 (3)
O1^ii^—Ni1—N1^ii^	97.72 (12)	C1—N1—Ni1	104.5 (3)
N1—Ni1—N1^ii^	83.58 (14)	C2—N1—Ni1	109.8 (3)
O1^i^—Ni1—N1^i^	97.72 (12)	C1—N1—H3	117.3
O1—Ni1—N1^i^	93.87 (12)	C2—N1—H3	101.4
O1^ii^—Ni1—N1^i^	177.00 (13)	Ni1—N1—H3	109.7
N1—Ni1—N1^i^	83.58 (14)	O2^iii^—N2—O2^iv^	119.999 (2)
N1^ii^—Ni1—N1^i^	83.58 (14)	O2^iii^—N2—O2	120.000 (3)
N1—C1—C2^i^	110.3 (4)	O2^iv^—N2—O2	120.000 (2)
N1—C1—H1A	109.6	Ni1—O1—H4B	117 (4)
C2^i^—C1—H1A	109.6	Ni1—O1—H4A	107 (4)
N1—C1—H1B	109.6	H4B—O1—H4A	104 (5)
C2^i^—C1—H1B	109.6		
			
C2^i^—C1—N1—C2	72.1 (5)	N1^ii^—Ni1—N1—C1	114.6 (2)
C2^i^—C1—N1—Ni1	−47.8 (4)	N1^i^—Ni1—N1—C1	30.4 (3)
C1^ii^—C2—N1—C1	−133.2 (4)	O1—Ni1—N1—C2	174.6 (3)
C1^ii^—C2—N1—Ni1	−16.3 (4)	O1^ii^—Ni1—N1—C2	89.3 (3)
O1—Ni1—N1—C1	−62.7 (3)	N1^ii^—Ni1—N1—C2	−8.1 (3)
O1^ii^—Ni1—N1—C1	−148.0 (3)	N1^i^—Ni1—N1—C2	−92.3 (2)

Symmetry codes: (i) *y*+1/2, −*z*+3/2, −*x*+2; (ii) −*z*+2, *x*−1/2, −*y*+3/2; (iii) *z*, *x*, *y*; (iv) *y*, *z*, *x*.

### Hydrogen-bond geometry (Å, °)

**Table:**

*D*—H···*A*	*D*—H	H···*A*	*D*···*A*	*D*—H···*A*
O1—H4A···O2^v^	0.84 (4)	1.95 (5)	2.776 (5)	162 (4)
O1—H4B···Br1^vi^	0.85 (5)	2.48 (5)	3.312 (3)	167 (4)

Symmetry codes: (v) −*x*+2, *y*−1/2, −*z*+3/2; (vi) −*x*+1, *y*+1/2, −*z*+3/2.
